# Framework for assessing bridge quality index based on the correlation of non-destructive test results

**DOI:** 10.1016/j.heliyon.2024.e26392

**Published:** 2024-02-15

**Authors:** Umesh Pant, Jagat Kumar Shrestha

**Affiliations:** Department of Civil Engineering, Pulchowk Campus, Institute of Engineering, Tribhuvan University, Nepal

**Keywords:** Bridges, Non-destructive tests, Bridge quality index, Deterioration level

## Abstract

This paper presents the new method for quality indexing of bridge components on the basis of non-destructive tests (NDT) for the assessment of the structural condition of Reinforced Concrete (RC) bridges. For the purpose of establishing indexing values, results obtained from the Electrical Resistivity Test (ERT), Rebound Hammer Test (RHT), Ultrasonic Pulse Velocity Test (UPVT), and visual inspection were used as the NDT variables. The radar plot for index determination is based on the area of the polygon whose vertices are formed on the basis of the correlation coefficient between NDT variables. Then, using the radar plot formulation, a bridge quality index is introduced to evaluate the corrosion and strength properties of in-service RC bridges to determine their structural health. The bridge quality index ranges from 0 to 1, representing low to high levels of deterioration. Based on simply non-destructive testing, the bridge quality index can serve as a framework for evaluating the present condition of the bridge and its components.

## List of symbols and abbreviations

NDT: Non-Destructive Test
RC: Reinforced Concrete
AASHTO: American Association of State Highway and Transportation Officials
UPE: Ultrasonic Pulse Echo
AADT: Average Annual Daily Traffic
IE: Impact Echo
GPR: Ground Penetrating Radar
IRT: Infrared Thermography
CR_VisInsp_: Condition rating by visual inspection
HCP: Half-Cell Potential
MATLAB: Matrix Laboratory
DOT: Department of Transportations
ERT: Electrical Resistivity Test
FWHA: Federal Highway Administration
$E_{d}$: Dynamic Modulus of Elasticity
$E_{c}$: Static Modulus of Rigidity
ASTM: American Society for Testing and Materials
UPVT: Ultrasonic Pulse Velocity Test
ERT: Electrical Resistivity Test
RHT: Rebound Hammer Test
$V_{p}$: Pulse Velocity

## 1 Introduction

Structural health monitoring of bridges using Non-destructive tests (NDTs) has not only wide applications due its non-damageable effect but also has received a grown attention in recent years due to the increased cost of repairs of degraded bridges (Akgul, 2021) [1]. Structural health monitoring system aims a purpose of developing a validation of design assumption and improvement in design guideline and specification of similar structures (Ko & Ni, 2005) [2]. In addition, they provide real-time information for safety assessment immediately after disasters and extreme events and provide evidence and lessons for planning and prioritizing bridge inspection, rehabilitation, maintenance and repair (Ko & Ni, 2005) [2]. A rating system of Federal Highway Administration (FHWA) in United States of America (USA) depicts the fact that 40% of the national bridges are deteriorated structurally which further leads to the conclusion that the average life span of the national bridges has also fallen to 42 years for those bridge whose design life span was at least 50 years (Pines & Aktan, 2002) [3]. A regular inspection system for condition assessment is, therefore, of vital importance for the prevention of any probable functional or structural deterioration. The effective use of NDT for the periodic inspection and evaluation of the condition of in-service bridges has become a daunting challenge due to the degree of effectiveness of the tests, proper quantification and correlation of the tests. In a survey questionnaire of Department of Transportation (DOTs) in different states and territory of USA, it has been found that only four NDT methods out of sixteen showed higher than 60% acquaintance or exposure rate in concrete bridges. Also, most of the NDT methods were considered difficult on the corresponding scale (Lee & Kalos, 2015) [4].

The research in structural health monitoring through NDT has been increasing recently. (Kim et al. (2022)) [5] conducted research to determine correlation between ultrasonic pulse velocity method and coarse aggregate for estimating residual modulus of elasticity of concrete exposed to high temperatures. Following a similar trend to the residual compressive strength, the ultrasonic pulse velocity and modulus of elasticity decreased as the temperature rose. They found that in comparison to normal concrete, light weight concrete has higher residual compressive strength, residual ultrasonic pulse velocity, and residual elastic modulus. A more advanced technique, Ground Penetrating Radar (GPR) was used to detect rebar in deck and quantification of deterioration through the use of scan of more than 4000 B-scan images (Asadi et al., 2019) [6].

The reliability of data obtained from different NDTs depends on the condition with which the test is done. Finalizing the output obtained from only one test or from a few sample points in the structural components can have a high chance of not showing the correct level of deterioration. So, coupling of various tests can help in getting actual conditions. Tests like hammer, chain drag, and coin tap tests are low-cost methods, but they are less reliable because they rely on human factor (Kashif Ur Rehman et al., 2016) [7]. The moisture content, homogeneity, and corrosion rate of reinforcing steel bars can be ascertained with electrical resistivity test (Polder et al., 2000) [8]. The challenging factor in it is temperature-related adjustments must be made to the data since a change in temperature of 3–5% per degree or a reduction of roughly 20 °C will result in a double resistivity value (Polder et al., 2000) [8]. For instance, in half cell potential test, more negative potential readings are typically thought to indicate a higher likelihood of corrosion, however this isn't always the case (Gu & Beaudoin, 2012) [9]. The measurements can be shifted towards positive and negative values by a variety of circumstances and half-cell data should only offer the likelihood of corrosion at a particular place during a certain test period. On the other hand, ongoing observation of this data is more beneficial (Gu & Beaudoin, 2012) [9]. Moreover, if the coupling issue is adequately fixed, the sonic and ultrasonic tests are more dependable and capable of addressing the greatest number of issues (Kashif Ur Rehman et al., 2016) [7].

The purpose of this work is to develop a bridge quality index by taking into account both the efficacy and interdependency of the results from several NDT test methods.

## 2 Non-destructive test (NDT) methods

### 2.1 Visual inspection test

For visible concrete surfaces, this test can be useful. To get the most information from the visual inspection, one must have a thorough understanding of structural engineering, concrete materials, and construction techniques (Davis et al. n. d.) [10]. Additional tools like handheld magnifier, stereo microscope, fiberscopes, and borescopes can be useful to observe a more detailed view of areas of cracks and distress (Kashif Ur Rehman et al., 2016) [7]. Despite being rapid and cheap to determine superficial problems in concrete, it never tells detailed and quantitative information about internal anomalies and defects (Kashif Ur Rehman et al., 2016) [7]. The fundamental drawback of visual examination is that it only captures cracks, degradation, and damage when they have just start to affect the life of the building or, in certain situations, after they have severely damaged the internal layers of the structure while only minor cracks are visible on the surface (*Reliability of Visual Inspection for Highway Bridges, Volume I: Final Report*, 2001) [11]. According to the Federal Highway Administration (USA), concrete bridges' average condition ratings were determined to be erroneous in at least 56% of cases, with 95% of the data coming from visual inspections (Pines & Aktan, 2002) [3]. Visual inspection can also be important and useful method to determine supplement NDT methods required to further investigate real condition of the structural components.

Based on the results obtained from visual inspection, the evaluation methods were developed (Akgul, 2013) [12]. A visual inspection condition rating system was developed (Akgul, 2021) [1] based on prioritization of the bridges depending on the condition rating of damage types (Akgul, 2015) [13]. The rating system as shown in Table 1 scales the bridge from one to four, one being the least deteriorated.

**Table 1:** Visual inspection condition rating scale (Akgul, 2021) [1].

Rating (CR_VisInsp_)	Description
1 (4 for radar plot)	No deterioration. (Like new condition)
2 (3 for radar plot)	Low deterioration (Good condition)
3 (2 for radar plot)	Medium deterioration (Satisfactory)
4 (1 for radar plot)	Heavy deterioration (Poor Condition)

Furthermore, it is recommended that this method be used in conjunction with other NDT procedures to ascertain the true condition of the structure.

### 2.2 Rebound hammer test

The rebound hammer test, being very simple and easy to use, has been used since 1950 (Lee et al., 2014) [4]. The Schmidt rebound hammer is primarily used to measure surface hardness. The compressive strength is affected by the water-cement ratio, voids present, the type of concrete it is intended for, the size of aggregate, the size of sand grains size and their distribution, admixtures added, and other cementing materials in concrete. It is seen that usage of finer sand enhances compressive strength (Tayehet al., 2022) [14]. It operates on the principle that the extent of rebound of the elastic mass depends on the hardness of the surface which it impacts. When the impact and rebound velocities (V_O_ and V_R_), are measured by the device, the basic parameter, the rebound value, is obtained from ratio of these values (Marić et al., 2019) [15]. There is no absolute scale for concrete strength based on the measured rebound due to the considerable heterogeneity of concrete mixes. Therefore, this method is limited to evaluating the relative concrete strength of a concrete bridge (Hartle et al., 2002) [16].

Correlation for compressive strength with rebound number of types Schmidt hammer (L) is given by (*The SilverSchmidt Reference Curve*, n. d.) [17] Equation (1) as:

(1) $$\text{fc}=1.9368e^{\left(0.0637Q\right)}\;N/{\text{mm}}^{2}$$

Where fc is the compressive strength and Q is the rebound hammer number.

### 2.3 Ultrasonic Pulse Velocity Test (UPVT)

The UPVT can be considered one of the oldest methods to assess the quality of concrete (Malhotra & Carino, 2003) [18]. This test measures velocity of ultrasonic pulses through concrete which helps to evaluate the structural integrity and strength of the concrete. This device has two transducers that transmit and receive the signal. The structural member, which is tested experiences a stress pulse that is generated by the transmitting transducer. The receiving transducer then receives the signal, and the outcomes are read as travel time. A little portion of the energy emitted is reflected back to the surface as the wave reaches out to a defect. As a result, compared to concrete in intact regions, the velocity of concrete will be substantially reduced in areas where there is significant degradation or micro cracking (Kashif Ur Rehman et al., 2016) [7]. The factor affecting ultrasonic wave transmission in concrete mainly corresponds to the types of cement, the positioning of transducers, age of the concrete, the distance between transducers, shape, and size of aggregates and admixtures (Ndagi et al., 2019) [19]. According to Hannachi &Guetteche (2012) [20], those with rounded aggregate edges provided lower propagation speeds than those with crushed aggregate. The degree of saturation as a parameter has also been found to affect the pulse speed by almost 4% (J.H. Bungey, 1984) [21]. There is a decrease in pulse velocities for regions with low compaction or voids which consequently indicates low water/cement ratio (Panzera et al., 2008) [3]. Further, the speed of pulse velocity reduces when waves come into contact with steel rebar more significantly when there is direct transmission (J.H. Bungey, 1984) [21].

**Fig. 1:**
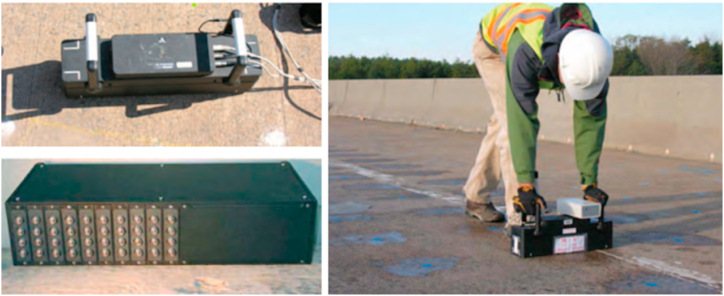
Bridge deck surveying using ultrasonic equipment (Gucunski et al., 2013) [24].

The pulse velocity test follows the principle that the density, homogeneity, and static modulus of elasticity of the material passing through it determine the pulse's ability to travel through any medium (Yaman et al., 2001) [22]. The guidelines provided in Table 2 can be used to evaluate the uniformity of concrete quality (Indian Standards, 1992) [23]. Fig. 1 illustrates the use of ultrasonic technology for bridge deck surveying.

**Table 2:** Velocity criterion for concrete quality grading as per IS 13311(Part 1): 1992 [23].

S·N.	Pulse velocity (km/s)	Quality grading
1	Above 4.5	Excellent
2	3.5 to 4.5	Good
3	3 to 3.5	Medium
4	Below 3.5	Doubtful

The dynamic Young's modulus of elasticity (E_d_) of the concrete may be determined from the $E_{d}=\frac{\left(1+\nu \right)\left(1-2\nu \right)\rho V^{2}}{\left(1-\nu \right)}$ (Indian Standards, 1992) [23], E_d_ = Dynamic Young's Modulus of elasticity in MPa ρ = density in kg/m^3^, And V = pulse velocity in m/second. The value of the dynamic Poisson's ratio varies from 0.20 to 0–35, with 0.24 as average (Indian Standards, 1992) [23]. The compressive strength and static modulus of elasticity can be determined using different literature mentioned in Table 3 and Table 4.

**Table 3:** Correlation to compressive strength fc from ultrasonic pulse velocity Vp.

S·N.	Compressive Strength (fc)	Quality grading
1	1.146exp (0.77Vp)	(Panzera et al., 2011) [25]
2	0.569exp (V_p_)	(Solís-Carcaño& Moreno, 2008) [26]
3	8.4 ∗ 10−9*(Vp)^2.5921^	(Kheder, 1999) [27]
4	0.8822exp (0.0008Vp)	(Bilgehan & Turgut, 2010) [28]

**Table 4:** Static modulus of elastic from dynamic elasticity value.

S·N.	Static Modulus of elasticity (E_c_) Gpa	Quality grading
1	0.83Ed	(Yaman et al., 2001) [22]
2	1.25Ed-19	(Standard, n.d.) [29]
3	1.04Ed - 4.1	(Standard, n.d.) [29]
4	$\frac{k{E_{d}}^{0.25}}{\rho }$	(Popovics et al., 2008) [30]

Ultrasonic pulse echo surveys are time-consuming because very close space must be left between test spots for images of the medium being tested to develop. The coupling of the sensor unit, which can be challenging on rough surfaces, has a significant impact on the data quality. As a result of the surface waves hiding the necessary compression or shear-wave signals, very shallow flaws may go undetected. Additionally, because it operates at lower frequencies, some faults could go unnoticed (Gucunski et al., 2013) [24]. This method is less effective for brittle materials, irregular shapes, or very thin materials (Lee et al., 2014) [4].

### 2.4 Half-cell potential measurement

**Fig. 2:**
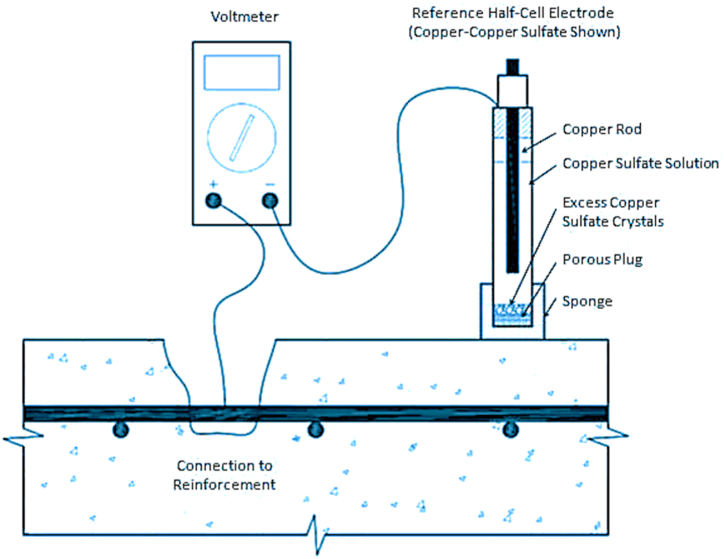
Apparatus of half-cell potential method (ASTM C-876-91, 1999) [32].

An established and popular electrochemical method for assessing active corrosion in reinforced steel and prestressed concrete structures is the half-cell potential (HCP) measurement. The technique is applicable in any environment and at any point in a concrete structure's lifespan as long as the temperature is higher than 2 °C (Elsener et al., 2003) [31]. Half-cell measurements should be performed on a concrete surface that is free of isolating layers (asphalt, coating, and paint), as these materials may cause measurements to be inaccurate or impossible (Gucunski et al., 2013) [24]. According to ASTM C876-91(ASTM C 876–91, 1999) [32], if the reinforcement is corroding, the electrons will have a tendency to travel from the reinforcement to the half-cell, where copper ions will be changed into copper atoms that will be deposited on the reinforcement. It would be highly likely that the reinforcement is corroding if the voltmeter showed more negative readings as shown in Table 5. The typical apparatus for half-cell potential measurement is shown in Fig. 2. During studies, it was found that the negative potential would increase if there were surface flaws such cavities (Kashif Ur Rehman et al., 2016) [7]. For bridge decks, a grid system is initially put out on the deck. Although 1.2 m is the ideal distance according to ASTM C876-91 (ASTM C 876–91, 1999) [32], a closer distance is advised if the voltage differential between the adjacent locations is greater than 150 mV.

**Table 5:** Criteria of ASTM C-876 on the corrosion probability in function of corrosion potential (ASTM C 876–91, 1999) [32].

S·N.	Cu/CuSO4 electrode (mV)	Corrosion Probability
1	More Positive than −200	Low (<10%)
2	Between −200 and −350	Intermediate
3	Between −350 and −500	High (>90%)
4	More negative than −500	Severe

To evaluate the corrosion activity on concrete bridges, the survey results can be generated as equipotential contour maps, which can then be used for maintenance and repair operations (McCann & Forde, 2001) [33]. Finally, the results can be assessed using the potential difference technique or a numerical approach as shown in Fig. 2. Irrespective of the thickness of the concrete cover and the size and details of the reinforcement, the half-cell potential approach can be used on concrete structures (Gu & Beaudoin, 2012) [9].

### 2.5 Electrical resistivity test

**Fig. 3:**
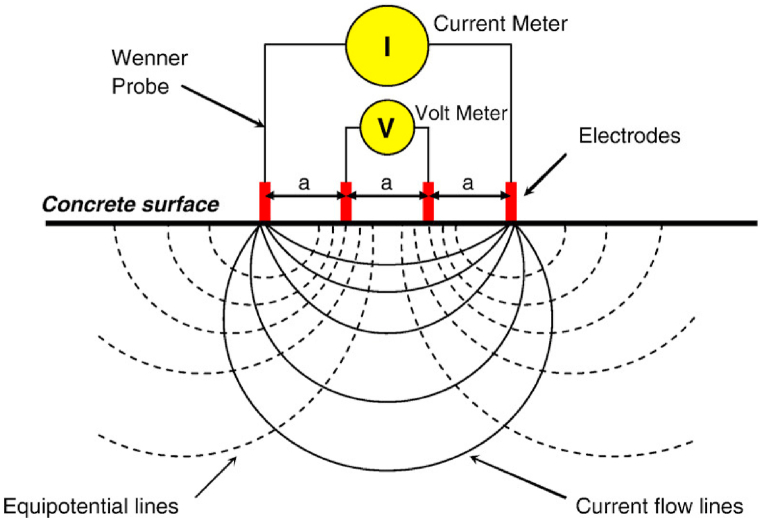
Schematic representation of ER measurement (Sbartaï et al., 2007) [36].

One of the most important durability performance indicators for assessing reinforcement corrosion in concrete is the surface electrical resistivity of the material. In addition to providing more details on the condition of the materials and the structure, it also makes it possible to estimate remaining life of structures (Andrade, 2018) [34]. Four electrodes are in contact with the concrete surface in the measurement equipment, which is based on the Wenner-probe technique with an alpha configuration (Garzon et al., 2014) [35] and schematic diagram is as shown in Fig. 3.

The two inner electrodes measure the electrical potential V created when the exterior electrodes apply an AC current I to the concrete. The apparent resistivity (ρ) in “ohm-m” may be expressed in Equation (2) as:

(2) ρ=2πaV/I

Where V is voltage drop Volts, I is applied current (amp), and a is electrode spacing (m) (Kashif Ur Rehman et al., 2016) [7].

Various Correlations between ER and probable corrosion are presented by literatures which are illustrated as in Table 6 and Table 7.

**Table 6:** Probable corrosion rate (Hornbostel et al., 2013) [37].

S·N.	Resistivity (Ω-m)	Likely corrosion rate
1	<50	VERY HIGH
2	50–100	HIGH
3	100–200	LOW
4	>200	NEGLIGIBLE

**Table 7:** Likely corrosion rate (Andrade, 2020) [38].

S·N.	Corrosion current (μA/cm^2^)	Likely corrosion rate
1	<0.1	PASSIVE
2	0.1 to 0.5	LOW – MODERATE
3	0.5 to 1	INTERMEDIATE
4	>1	HIGH

Climate conditions have an impact on the values found in actual structures, necessitating a careful analysis of all three parameters-potential, resistance, and corrosion rate (Andrade, 2020) [38]. According to the literature, combining ER with an acoustic method and other NDT methods will result in an accurate diagnosis (Kashif Ur Rehman et al., 2016) [7].

The Pros and Cons of NDT methods are summarized in Table 8.

**Table 8:** Pros and Cons of NDT methods used.

NDT Methods	Applications	Limitations	Key references
UPVT	Detect cracks, depth and locate abnormal region in concrete.	Require narrow grid spacing, less effective for brittle materials, difficult on rough surface	(Sack & Olson, 1995) [39] (Hartle et al., 2002a) [16]
Half-cell method	Used to detect corrosion, can be used at any time or climatic condition	Minimum temperature should be −2° Celsius, determines probability of corrosion, data collection not possible if there is coating (asphalt or paint) concrete	(Polder et al., 2000) [8] (Hartle et al., 2002a) [16] (Gu & Beaudoin, 2012) [9]
ERT	Detect cracks delamination and proneness to corrosion	Surface should be pre wetted, ER is affected by moisture and salt content, porosity	(KashifUr Rehman et al., 2016) [7] (Polder et al., 2000) [8]
Rebound hammer test	Cheap and simple to use and calculate the strength, asses the strength of old structures	Calibration for varying concrete types and aggregate types should be done, Requires core samples for reference, difficult for overlays	(Hartle et al., 2002) [16]
GPR	Delamination, concrete thickness, rebar configuration and embedment depths, bridge deck condition, contour maps of subsurface features	Not suitable as thickness of increases, Expensive methods, Difficult in extreme cold condition, unable to determine the strength	(Kashif Ur Rehman et al., 2016) [7](Hartle et al., 2002) [16]
Impact echo	Detect defects, delamination, wave deflector, voids, modulus of elasticity evaluation	Multiple impact location for high accuracy, Dense grids required, difficult in overlays	(Kashif Ur Rehman et al., 2016) [7] (Hartle et al., 2002) [16]
Acoustic emission	Real time image detection, detects dislocations, no interference to traffic flow	No standard process, Sensitive to external noise, less effective for certain loading combination	(Kashif Ur Rehmanet al., 2016) [7] (Lee & Kalos, 2015) [40]

## 3 Methodology

The NDT variables are normalized or squeezed between 0 and 1 or -1 to 1 for determining the quality index which varies to the value 1. There are various types of squeezing or squashing mathematical logistic functions like sin(x), sigmoid function, arctan function, hyperbolic tangent function, etc. However, all these functions are not increasing function except the sigmoid function. For an increasing squashing function in the domain of real numbers, the sigmoid function is chosen as it is increasing, has domain (-∞, ∞), and has higher sensitivity towards lower values.

### 3.1 Sigmoid function

The sigmoid function can map the input signal between 0 and 1 in a straightforward manner and is continuously differentiable throughout the entire function domain. The function is given as in Equation (3). Given that its domain is the set of all real numbers, and its range is (0, 1), the sigmoid function is also known as a squashing function. As a result, the output is always between 0 and 1 regardless of whether the function is given a very large positive or very large negative number as input (Saeed, 2021) [41]. The output or readings obtained from NDTs, which ranges between two specific values other than range starting from zero is squashed using sigmoid function. The reading that ranges from 0 to positive maximum value is squashed from modified sigmoid function as in Equation (4).

(3) $$\text{Sigmoid}\;\text{function}\;f\left(x\right)=\frac{1}{1+e^{-x}}$$

(4) $$\text{Sigmoid}\;\text{function}\;f\left(x\right)=\frac{1-e^{-x}}{1+e^{-x}}$$

**Fig. 4:**
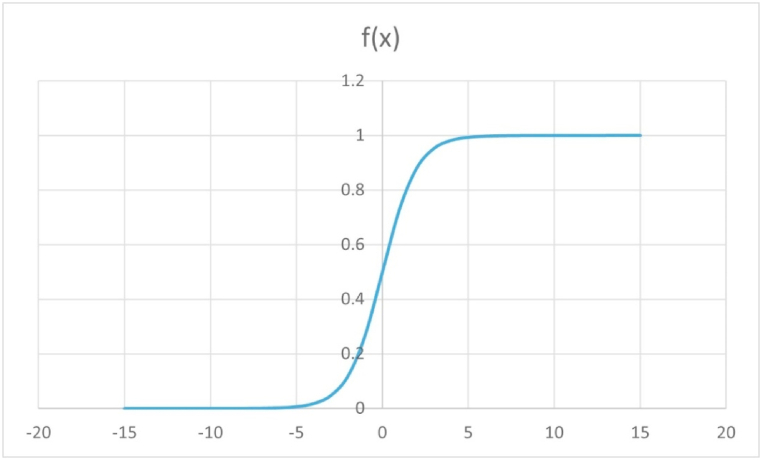
Plot of sigmoid function $\frac{1}{1+e^{-x}}$:

**Fig. 5:**
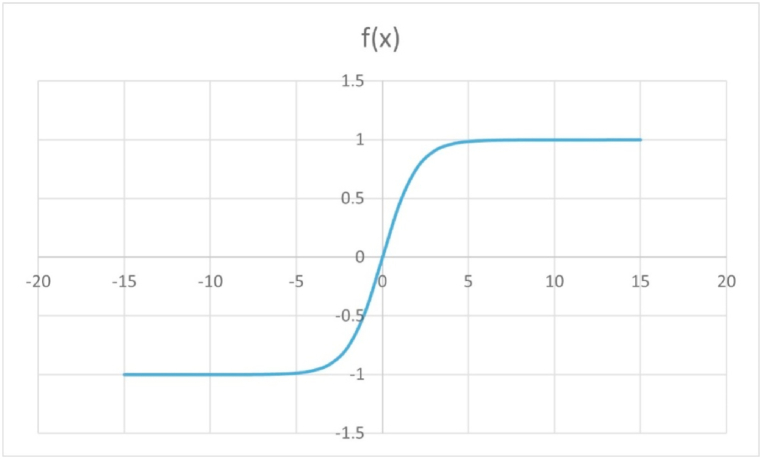
Plot of modified sigmoid function $\frac{1-e^{-x}}{1+e^{-x}}$:

The choice of the above-mentioned sigmoid functions is due to the reasons that this function constricts our range within [0, 1] while the domain is (-∞, ∞)for the sigmoid function. The graph of the sigmoid function and its modified form are depicted in Fig. 4, Fig. 5. The modified sigmoid function is modified and applied for the range of NDT variables whose least value is zero, so that the corresponding squashed value is also zero. For higher values, it gets saturated to a maximum of 1. For values having minimum values other than zero or for the NDT variables whose lower value has the best quality in terms of strength or deterioration the sigmoid function is used as described in section 3.2.1.

These functions are continuously increasing function, and they are sensitive to lower values. The sigmoid function chosen for the positive range value or upper range is considered at 95% saturation level. For instance, from Table 5 best value of corrosion potential is −200mV which infer that reading more than −200mV are best and get saturated, but for the lower values near to −500 mV, it is highly sensitive.

### 3.2 Formulation of indexing radar plot

#### 3.2.1 Squashing the obtained NDT variables

Firstly, the readings from NDTs are squashed from 0 to 1 based on the best value of saturation which is shown in Table 9. The squashing function depends on the type and range of value.

**Table 9:** NDT methods with variables value range for squashing.

S·N.	NDT methods	NDT variable	Best Value	Worst value	Squashing equation
1	Rebound Hammer Test	fc/fcp (Hadianfard et al., 2017) [42]	1	0	Modified Sigmoid Function (N)
2	Ultrasonic Pulse Velocity Test	Ec/Ecp (Hadianfard et al., 2017) [42]	1	0	Modified Sigmoid Function (N)
3	Electrical resistivity Test	Ρ (Ω-m) (Table 6)	200	0	Modified Sigmoid Function (N)
4	Half-cellpotential test	Electric Potential (mV) (Table 5)	−200	−500	Sigmoid Function (E)
5	Electrical resistivity Test	Corrosion Current (μA/cm^2^) (Table 7)	0.1	1	Sigmoid Function Reverse (R)
6	Visual Methods	Rating (Akgul, 2021) [1]	5	1	Sigmoid Function (E)

For example, for corrosion current the higher value of corrosion current resembles a high corrosion rate and vice versa (type R). This type of data is considered as reverse type and squashed using the sigmoid function. The normalized values obtained are subtracted by one to show a value when the normalized value is tending to one. Similarly, the ratio of in-service condition and initial condition of compressive strength (fc/fcp) and static modulus of elasticity (Ec/Ecp) range from 0 to 1. This type of data is considered as type N and normalized by a modified sigmoid function. The remaining range between two specific values is considered as type E and squashed by the sigmoid function.

#### 3.2.2 Correlation and plotting of NDT variables

**Fig. 6:**
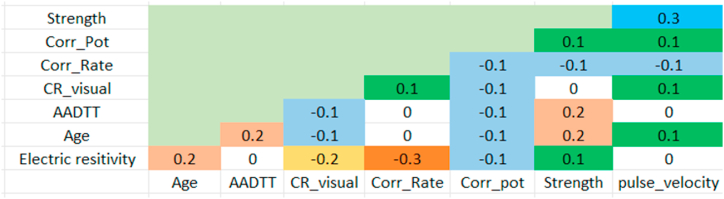
Correlogram showing the correlations and independence among NDT variables (Akgul, 2021) [1].

Searching for correlations and statistical independence among NDT variables yields detailed results that are displayed as a matrix of scatter plots in research taken in 100 bridges in Turkey. Equations for a linear regression model have been created based on the correlation cases (Akgul, 2021) [1]. Additionally, the correlation between various NDT measurements was examined, and the relationships exhibiting standout correlations were presented as depicted in Fig. 6.

Plotting of NDT variables to determine the quality index of bridge elements is based on the correlation of different tests. The process employed is illustrated as.

1. The best two correlated test variables that can form adjacent vertices of the polygon in the radar plot were selected.
2. Then, the next best-correlated values for the first two values already selected were determined.
3. Step 2 is further repeated, and the repeated test variables are cancelled out as they already appeared.
4. The chain with the highest correlated coefficient is followed and the chain to form a polygon is completed.
5. The angle between two NDT variables is inversely related to the correlation coefficients i.e., if the test variables are perfectly correlated plotting of only one test variable is enough. Consequently, higher correlated NDT variables form the smaller interior angle i.e., an interior angle is proportional to (1-$r^{2}$) where r is the correlation coefficient. Thus, the angle between two adjacent variable line can be given by Equation (5) as:

(5) $$\theta_{m}=\frac{360\left(1-r_{m}^{2}\right)}{\sum_{i=1}^{i=n-1}\left(1-{r_{i}}^{2}\right)}$$

For example, if there are three NDT variables corrosion rate, electrical resistivity and visual inspection, the correlation as 0.4 between corrosion rate and electrical resistivity, 0.2 between electrical resistivity and visual inspection and 0.1 between electrical resistivity and visual inspection, the angles can be calculated using Equation (5) as follows:

The highest two correlated NDT variables are corrosion rate and electrical resistivity and hence they form two adjacent vertices and the angle is 108.387^0^. The next highest correlated variable to electrical resistivity is visual inspection whose interior angle from the center becomes 123.87^0^ and the third angle between visual inspection and corrosion rate is calculated as 127.741^0^.

**Fig. 7:**
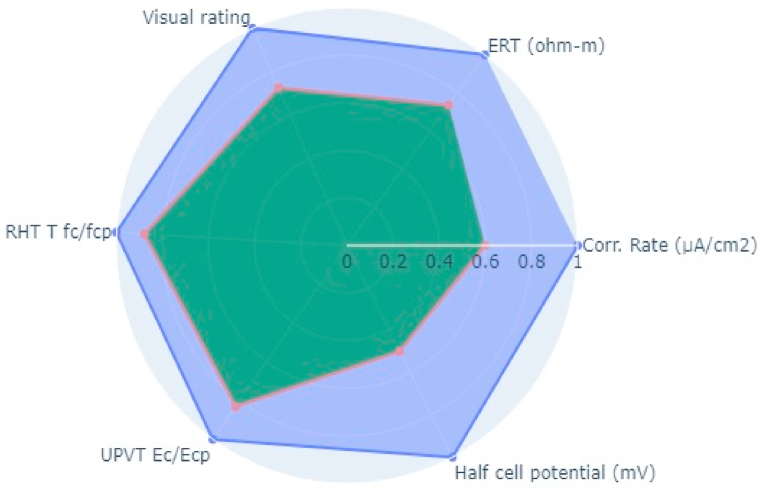
Sample plot for indexing of bridge components (Index = 0.51).

6. Finally, the polygons plotted were obtained from programming in Python as shown in Fig. 7 using variables mentioned in Table 9 (arbitrary values of resistivity = 103 Ω-m, visual rating = 3, fc/fcp = 0.75, Ec/Ecp = 0.65, potential = −350, corrosion rate = 0.2).

#### 3.2.3 Bridge element quality indexing

All the normalized values are plotted for maximum value to form a regular polygon of maximum area. Once squashed values of NDT variables are found, distance from the center point is determined and the corresponding coordinates (x,y) are calculated using trigonometry. The coordinated formula for calculating areas as $Area=0.5(ABS(\sum_{i=1}^{n-1}\left(\left(x_{i}y_{i+1}-x_{i+1}y_{i}\right)+x_{n}y_{1}-x_{1}y_{1}\right)$))) where n is the number of vertices of the polygon. As seen in Fig. 7, which shows blue as the standard polygon and green as the actual status of the bridge component, each vertex of the polygon represents the highest value of one in the polygon, and the centroid of the polygon represents the zero value for each of the NDT variables.

The ratio for the area of a bigger polygon to a smaller polygon is the quality index of the respective bridge representing quality degradation with respect to strength, corrosion, and elasticity values which is depicted in Table 12.

**Table 10:** Normalized parameters fc/fcp, Ec/Ecp, ρ along with bridge quality index atDhobikhola and Hanumante bridge.

DHOBIKHOLA BRIDGE					HANUMANTE BRIDGE				
Test points	RHT	UPVT	ERT	**Bridge Deck**	Test points	RHT	UPVT	ERT	**Bridge Deck**
				**Quality index**					**Quality index**
	fc/fcp	Ec/Ecp	Ρ			fc/fcp	Ec/Ecp	Ρ	
1	0.81	1.07	183	0.87	1	0.61	0.7	87	0.6
2	0.87	1.11	222	0.9	2	0.45	0.61	81	0.49
3	0.5	0.63	194	0.68	3	0.41	0.57	107	0.52
4	0.73	0.78	153	0.78	4	0.59	0.99	73	0.59
5	0.67	0.95	124	0.75	5	0.54	0.57	64	0.47
6	0.7	0.97	86	0.66	6	0.5	0.68	121	0.63
7	0.84	1.2	96	0.74	7	0.43	0.55	67	0.43
8	0.4	0.82	99	0.56	8	0.5	0.57	71	0.47
9	1.09	0.82	131	0.81	9	0.55	0.6	124	0.63
10	0.75	0.95	134	0.79	10	0.59	0.65	57	0.48
11	0.65	0.89	160	0.78	11	0.63	0.73	67	0.55
12	0.68	0.81	171	0.79	12	0.66	0.45	86	0.52
13	0.95	1.06	146	0.85	13	0.58	0.64	90	0.58
14	0.94	0.85	101	0.74	14	0.66	0.57	132	0.67
15	**0.91**	**0.97**	**57**	**0.59**	15	0.52	0.54	51	0.4
16	0.61	0.95	158	0.77	16	0.57	0.98	78	0.6
17	0.62	0.82	263	0.81	17	0.64	0.61	56	0.48
18	0.56	0.77	150	0.72	18	0.72	0.38	66	0.44
19	0.49	0.85	138	0.68	19	0.61	0.98	138	0.75
20	0.66	1	95	0.68	20	**0.66**	**0.43**	**45**	**0.38**
21	0.71	0.93	99	0.7					
22	0.78	1.07	166	0.85					
23	0.69	0.82	119	0.72					
24	0.59	0.87	164	0.76

**Table 11:** NDT parameter based on data collected in bridge components in Croatia (Marić et al., 2019) [15] and corresponding quality indexing.

Sn.	Bridge	RHT fc/fcp	Electrical resistivity ohm-m	Half cell potential (mV)	Quality indexing
1	Adriatic bridge (AB3) Abutment wing moderate	1.25	203	−390	0.51
2	Adriatic bridge (AB5) girder	2.46	161	−215	0.89
3	Homeland bridge (deviator)	1.19	539	−70.25	0.98
4	Bridge of youth BY1 girder	1.88	69	−400	0.33
5	Bridge of youth BY2 girder	2.69	141	−144	0.9
6	Bridge of youth BY3 Abutment wall 2	1.32	123	−492	0.3
7	Bridge of youth BY4 Abutment wall 2	1.04	142	−549	0.29
8	Meslenica bridge MB1	0.58	21	−657	0.05
9	Pag bridge arch 1 PB1	0.23	69	−331	0.26
10	Pag bridge arch 2 PB2	0.58	13	−410	0.1

**Table 12:** Quality indexing and corresponding deterioration Level.

SN.	Index Value Range	Deterioration Level
1	0–0.25	High Deterioration
2	0.26–0.5	Medium Deterioration
3	0.51–0.75	Low Deterioration
4	0.76–1	Negligible Deterioration

## 4 Results

### 4.1 Numerical example for indexing of bridge deck

**Fig. 8:**
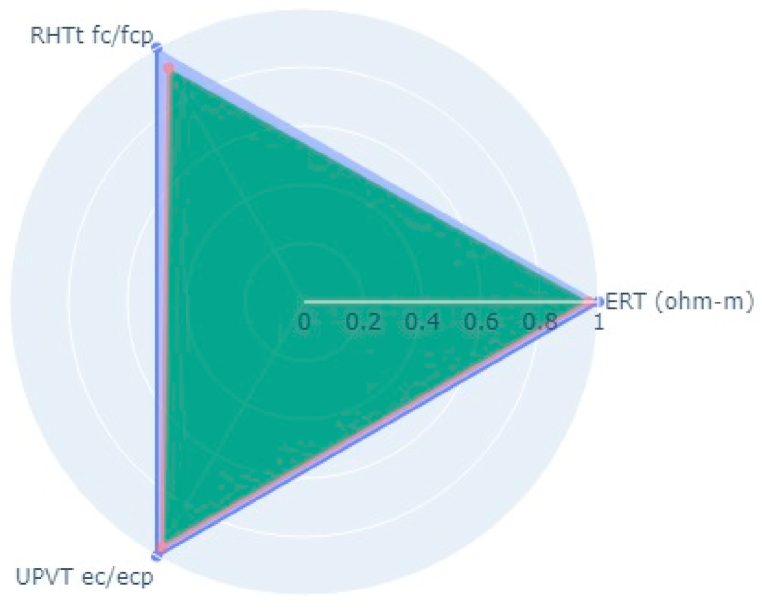
Maximum value of index at NDT point 2, index = 0.9 Dhobikhola bridge.

**Fig. 9:**
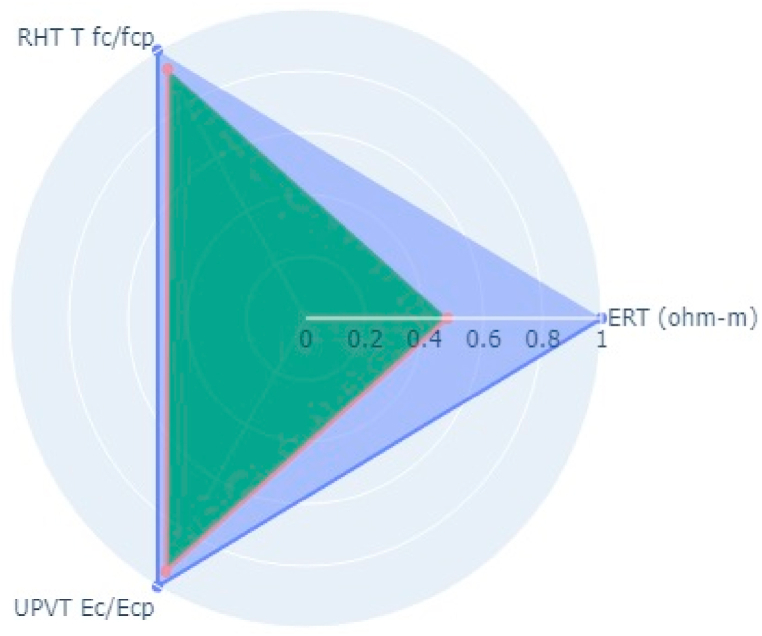
Minimum value at NDT point 15, index = 0.59 at Dhobikhola bridge.

**Fig. 10:**
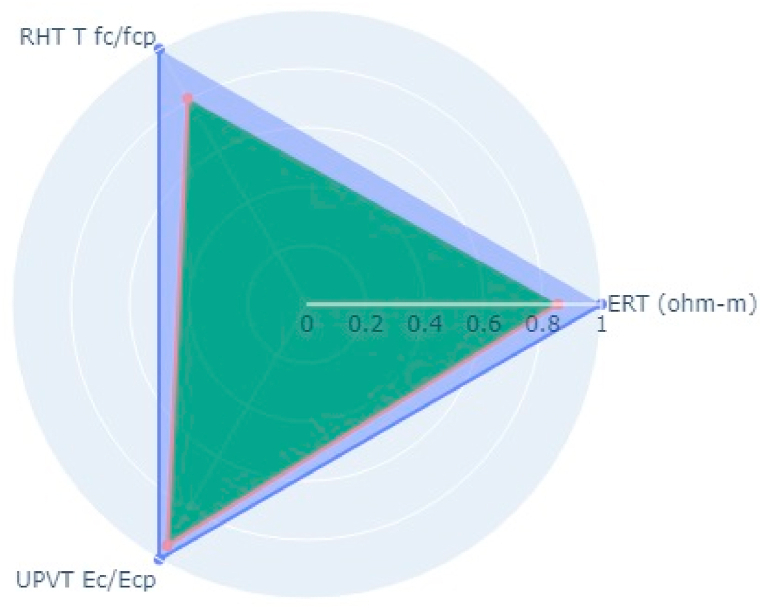
Maximum value at NDT point 19, index = 0.75 at Hanumante Bridge.

**Fig. 11:**
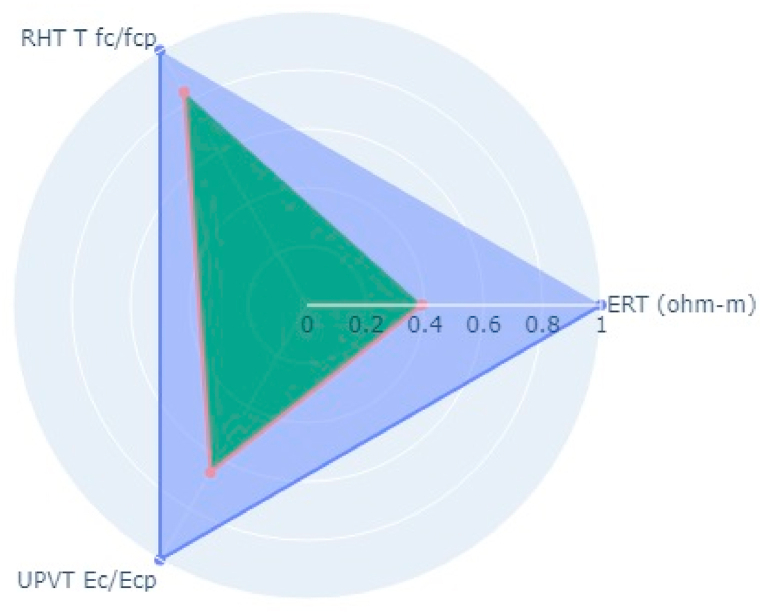
Minimum value of index at NDT point 20, index = 0.38 at Hanumante bridge.

Following the aforementioned methodology, NDT on two bridge decks in the Kathmandu (Dhobikhola Bridge) and Lalitpur (Hanumante Bridge) districts of Nepal were carried out (Pant & Shrestha, 2022). Rebound Hammer Test (RHT), Ultrasonic Pulse Velocity Test (UPVT), and Electrical Resistivity Test (ERT) were taken which are equally correlated with a coefficient value of 0.1. The NDT variable parameter to be fitted in the radar plot polygon with indexing of different points on the bridge deck is given in Table 10. The test was conducted on several points on the basis of visual inspection where the concrete was more degraded and where it was very prone to deterioration. The structural drawing of bridges was collected from the Department of Roads, Nepal and the reinforcement details were observed and studied. Based on the visual cracks, position of maximum loading and accessibility in the bridge, the bridge was divided into grids along the centre line along the longitudinal section. Along each cross-section, three tests were carried out. On Dhobhikhola bridge deck,at NDT point 2, maximum indexing of 0.9 was seen (Fig. 8), whereas, at NDT point 15, minimum indexing of 0.59 (Fig. 9) was seen which depicts satisfactory condition of the deck. On HanumanteBridge deck, at NDT point 19, maximum indexing of 0.75 was seen (Fig. 10) and at NDT point 20, minimum indexing of 0.38 (Fig. 11) was seen which depicts a satisfactory condition of the deck.

Based on the conducted test, the Hanumante bridge deck has a significant reduction of strength, and the reinforcement is highly susceptible to corrosion internal crack depth are prone to increase as almost half of the test points show indexing less than 0.5 and the remaining test point indexed were in the range 0.5–0.75 only, where the condition of the deck is comparatively of high quality in terms same parameters. On the other hand, the test on the Dhobikhola bridge deck showed an index value of more than 0.75 in more than 50% of points and more than 0.6 in all the test points, which suggests excellent condition of the bridge deck having negligible strength, minor cracks and condition of reinforcement almost free of corrosion.

### 4.2 Validation

**Fig. 12:**
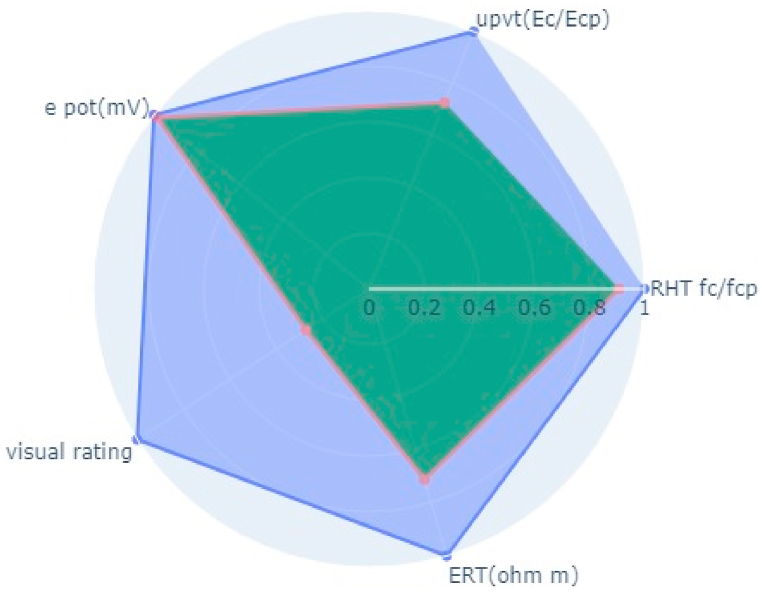
Bridge indexing (0.496) from bridge from (Akgul, 2021) [1].

For the validation of the framework mentioned earlier, research on 100 bridges in Turkey was done (Akgul, 2021) [1]. One of the bridges mentioned in the paper, the NDT variables as pulsevelocity, V_corrected_ = 3240 m/s (Indian Standards, 1992) [23], fc = 19.92 MPa, E_corr_ = −132.6 mV, ρ = 97.8 Ωm, visual rating = 3 (2 for radar plot). The rating of the bridge was found to be 2.85 as per the paper which was considered a low to medium level of deterioration in terms of strength and corrosion characteristics. These data of NDT variables are plotted in this radar plot of the mentioned methodology and the Bridge deck quality index was found to be 0.496 (Fig. 12) which concords with the literature (Akgul, 2021) [1]. This indexing signifies the bridge element has a high susceptibility to corrosion and load-carrying capacity is also reduced to some extent.

For further validation, research in Croatia bridges for the assessment of reinforcement corrosion and concrete damages was taken (Marić et al., 2019) [15]. On the basis of data collected by various NDT methods (Electrical resistivity test, rebound hammer test, and half-cell potential test) on different components of the bridge, the quality indexing was done.

**Fig. 13:**
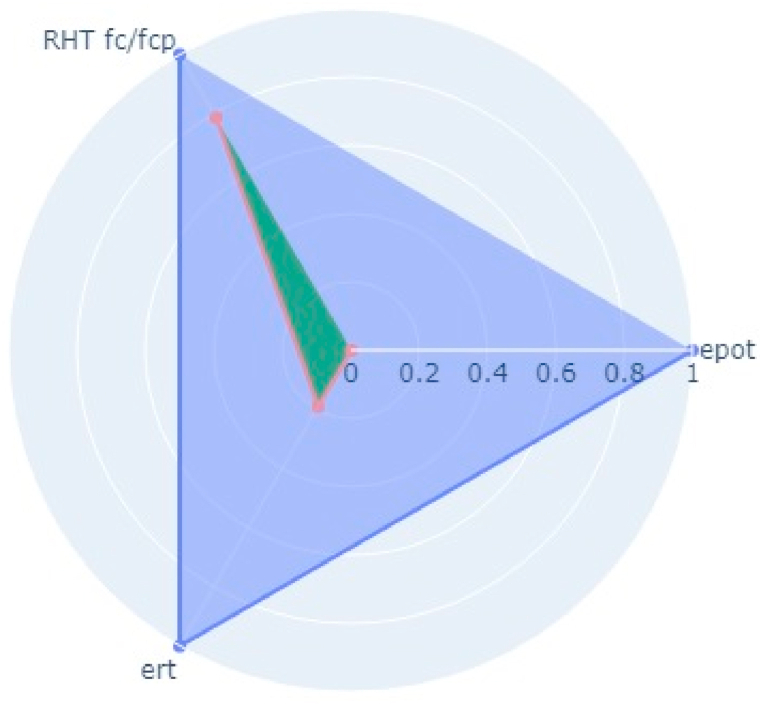
Bridge indexing of Meslenicabridge (0.05) bridge from (Marić et al., 2019) [15].

**Fig. 14:**
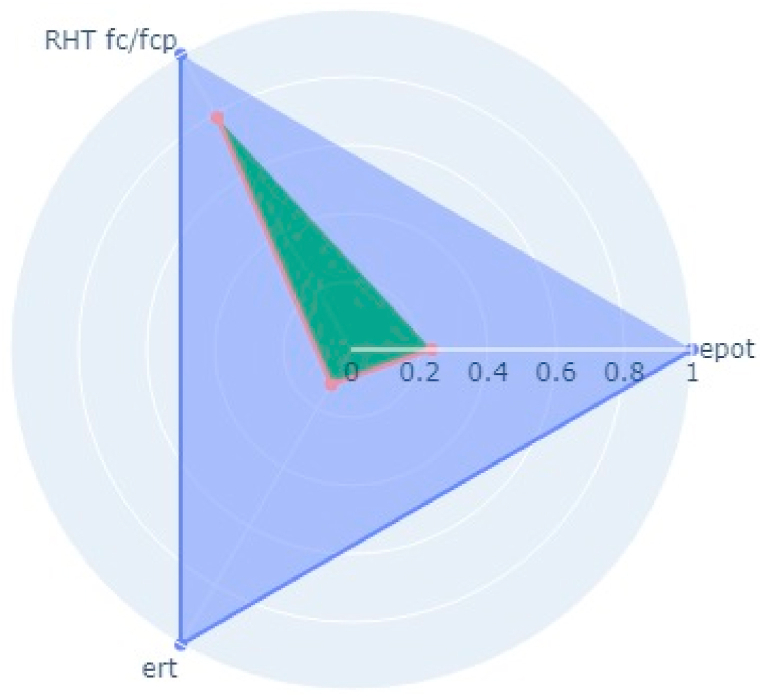
Bridge indexing of from Pag bridge arch 2, indexing = 0.1 (Marić et al., 2019) [15].

**Fig. 15:**
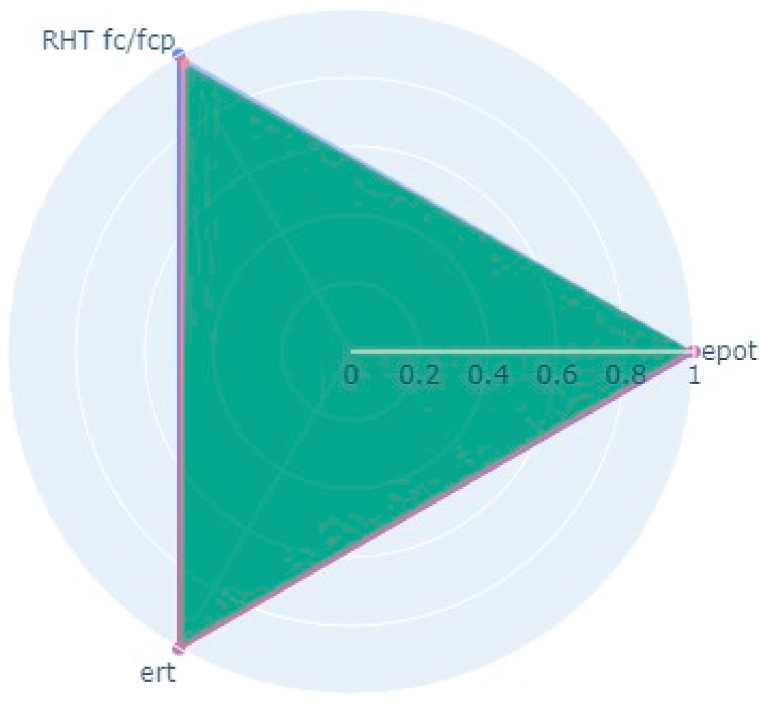
Bridge indexing of Homeland bridge (0.98) from bridge from (Marić et al., 2019) [15].

The paper indicated a very high corrosion rate and degradation (Marić et al., 2019) [15] in the Meslenica bridge and Pag Bridge arch 2 which can be seen in very low-quality indexing of 0.05 and 0.1 from Fig. 13, Fig. 14 (Table 11). Also (Marić et al., 2019) [15], say the best condition bridge is the Homeland bridge which is supported by the indexing of 0.98 (Table 11, Fig. 15).

Further, Pag Arch Bridge 1 having an index of 0.26, and Bridge of Youth BY4 Abutment wall 2 having an index of 0.29 as depicted in Table 11 has been considered to be medium deterioration (Marić et al., 2019) [15]. On this basis, the quality index between 0.25 and 0.5 has been categorized as medium deterioration as illustrated in Table 12.

The deterioration can be categorized into four levels High, Medium, Low, and Negligible deterioration levels as shown in Table 12. An index value of 0–0.25 indicates a high degree of deterioration, which includes deeper cracks, a considerable reduction in the concrete's ability to support loads, a higher amount of voids in the concrete, and a higher susceptibility to steel corrosion. A medium level of degradation, with a considerable number of cracks and a high sensitivity to reinforcing corrosion with corrosion start, is indicated by an index value in the range of 0.26–0.5. It also shows that small fractures and voids in the concrete contribute to its decreased strength. A low deterioration level, indicated by an index value of 0.51–0.75, indicates the presence of some surface cracks, some shallow cracks that have started, and some mild reinforcement corrosion. The load-carrying capability has only been slightly impacted. A low degree of deterioration is indicated by an index value of 0.76–1; there are only little surface cracks and no sign of rebar corrosion, and the strength of the concrete is not reduced.

## 5 Conclusion

This study correlated the data from the Rebound Hammer, Electrical Resistivity, and Ultrasonic Pulse Velocity NDT tests into the introduction of the Bridge Quality Index, taking into account the effectiveness of the test results as well as their interdependency. A framework for assessing bridge elements has been developed and can be used as a quality index for bridges and their components based on non-destructive tests. The application of NDT enhances the objectivity and precision of visual inspection results and makes it possible to detect hidden defects, notwithstanding the measurement accuracy limitations of each technique. The higher number of test variable means a higher number of sides in the polygon and consequently brings higher confidence to the quality indexing. Therefore, to increase the precision of the index values, NDT variables can be expanded by utilizing cutting-edge NDT technologies such as impact echo tests, acoustic emission tests, ground penetrating radar (GPR), and infrared thermography. The findings of the research offer the foundation for future advancements.

## Data availability statement

The data that support the findings of this study are available on request from the corresponding author.

## CRediT authorship contribution statement

**Umesh Pant:** Writing – original draft, Validation, Methodology, Investigation, Formal analysis, Data curation, Conceptualization. **Jagat Kumar Shrestha:** Writing – review & editing, Validation, Supervision, Methodology, Investigation, Formal analysis, Conceptualization.

## Declaration of competing interest

The authors declare that they have no known competing financial interests or personal relationships that could have appeared to influence the work reported in this paper.
